# Low-dose ventricular radiotherapy in wild-type transthyretin cardiac amyloidosis: a prospective, first-in-human, exploratory clinical trial

**DOI:** 10.1016/j.ijcha.2026.101909

**Published:** 2026-03-24

**Authors:** Damien Guijarro, Emmanuelle Massie, Thomas Zilli, Anna Beale, Valentina Garibotto, René Nkoulou, Philippe Meyer

**Affiliations:** a Service of Cardiology, Geneva University Hospitals, Geneva, Switzerland; b Radiation Oncology, Oncology Institute of Southern Switzerland (IOSI), EOC, Bellinzona, Switzerland; c Facoltà Scienze Biomediche, Università della Svizzera Italiana (USI), Lugano, Switzerland; d Faculty of Medicine, Geneva University, Geneva, Switzerland; e Division of Nuclear Medicine and Molecular Imaging, Geneva University Hospitals, Geneva, Switzerland; f CIBM Center for Biomedical Imaging, Geneva, Switzerland

**Keywords:** Cardiac amyloidosis, Transthyretin, Radiotherapy, amyloid PET

## Abstract

**Background:**

Wild-type transthyretin cardiac amyloidosis (ATTRwt-CA) causes heart failure through myocardial deposition of misfolded transthyretin (TTR) fibrils. Radiotherapy has been explored in localized amyloid deposition in other organs, but its potential role in ATTRwt-CA remains unexplored.

**Methods:**

Eligible patients with ATTRwt-CA underwent low-dose radiotherapy (LD-RT) (10 Gy in 5 daily fractions) targeting the left ventricle. Cardiac amyloid burden was assessed using 18F-Flutemetamol amyloid PET (tissue-to-background ratio (TBR) in the septal and lateral LV walls) and cardiac magnetic resonance (CMR) imaging (extracellular volume and T1 mapping) at baseline and 12 weeks. Additionally, New York Heart Association (NYHA) class, cardiac biomarkers, transthoracic echocardiography, 6-minute walk test (6MWT) and the Short-Form 36-Item Health Survey (SF-36) were evaluated at baseline and at weeks 3, 6, 12, and 6 months post-LD-RT. Safety was assessed through systematic clinical follow-up and monitoring of patient-reported symptoms potentially attributable to LD-RT.

**Results:**

Five patients with ATTRwt-CA (mean age 87 years) received focused LD-RT; two received concomitant tafamidis. No grade ≥ 3 treatment-related adverse events occurred over 6 months. At 12 weeks, clinical, biomarker, and functional changes were heterogeneous. A directional decrease in amyloid PET uptake ratio was observed in most patients, irrespective of tafamidis exposure, whereas native T1 values and left ventricular mass index on CMR showed no improvement.

**Conclusion:**

In this small exploratory cohort, cardiac radiotherapy was well tolerated. Although no efficacy conclusions can be drawn, the observed PET signal warrants cautious evaluation in adequately powered studies.

## 1 Introduction

Wild-type transthyretin cardiac amyloidosis (ATTRwt-CA) is an increasingly recognized, progressive infiltrative cardiomyopathy resulting from the accumulation of misfolded transthyretin (TTR) fibrils in the myocardial extracellular space, leading to heart failure and reduced survival. Until recently, tafamidis was the only approved therapy for ATTRwt-CA, following the pivotal ATTR-ACT trial published in 2018, which demonstrated its ability to slow disease progression and improve survival and quality of life [1]. More recently, acoramidis, another transthyretin stabilizer, has also shown clinical efficacy [2]. In parallel, gene-silencing therapies have improved clinical outcomes by reducing TTR production [3]. However, while these agents slow disease progression, they do not directly target or remove existing myocardial TTR deposits. Novel therapeutic strategies aimed at amyloid clearance, including the recombinant human anti-ATTR monoclonal IgG1 antibody NI006 and coramitug, have shown encouraging early-phase results in promoting myocardial TTR removal [4], [5]. However, the anticipated high cost of these therapies may restrict their accessibility, underscoring the ongoing need for novel, affordable strategies capable of depleting cardiac TTR deposits.

Radiotherapy (RT) has been successfully used for over two decades to treat extracardiac amyloidosis in selected symptomatic disease sites, including the tracheobronchial tree [6], [7], [8], skin [9], orbital and periorbital regions [10], [11] and bladder [12]. RT can potentially reduce the burden of amyloid deposits with a sustained response, although the underlying mechanism remains unclear. Analogous findings have been reported in Alzheimer’s disease, another amyloid-associated condition characterized by amyloid-β accumulation and tau neurofibrillary tangles, where preclinical studies and early human investigations have suggested a potential modulatory effect of low-dose radiotherapy (LD-RT) [13], [14]. Despite concerns about long-term side effects, the poor prognosis of ATTRwt-CA and the limited accessibility of emerging therapies support the exploration of LD-RT as a potential therapeutic approach [15].

This study aims to assess the safety and short-term effects of LD-RT on cardiac TTR amyloid deposits, as assessed by amyloid positron emission tomography (PET) with 18F-Flutemetamol and its impact on functional assessment, cardiac magnetic resonance (CMR) and echocardiographic parameters, cardiac biomarkers, and quality of life.

## 2 Methods

This was a non-randomized, open-label, uncontrolled exploratory before-and-after study conducted prior to the widespread availability of tafamidis.

Consecutive patients with ATTRwt-CA aged >65 years with symptomatic heart failure (NYHA ≥ II), positive DPD scintigraphy (Perugini II–III), compatible cardiac imaging, and baseline 18F-amyloid PET (TBR > 1.45) were eligible. Diagnosis followed current guidelines, with exclusion of hereditary forms by genetic testing. Major exclusion criteria included monoclonal gammopathy, prior thoracic radiotherapy, severe renal impairment, non–CMR-compatible devices, and active malignancy (full criteria in Supplementary Data).

LD-RT (10 Gy in five daily 2-Gy fractions) was delivered to the left ventricle (LV) using volumetric modulated arc therapy with daily image guidance [16]. The target volume, defined on 4D-CT, encompassed the LV excluding the basal halves of the antero- and inferolateral segments; a 3-mm margin generated the planning target volume (PTV). Target coverage required ≥ 98% of the PTV to receive ≥ 95% of the prescribed dose. Organs at risk were contoured per Radiation Therapy Oncology Group guidelines, with predefined dose constraints (mean heart and planning risk-volume of the left anterior descending artery < 5 Gy; lung < 7 Gy; esophagus < 5 Gy).

Efficacy was assessed at 12 weeks by amyloid PET, CMR, echocardiography, cardiac biomarkers, NYHA class, 6MWT, and SF-36 (Supplementary Table S1). Patients were followed at weeks 3, 6, 12, and 6 months. Safety monitoring included clinical assessment, serial biomarkers, ECG surveillance, and echocardiography, with adverse events graded according to the National Cancer Institute Common Terminology Criteria for Adverse Events (CTCAE v4) and predefined stopping rules overseen by an independent Data Safety Monitoring Board (see Supplemental data).

Based on preliminary PET data, 36 patients were initially estimated to provide adequate power [17]. However, only five were enrolled, and analyses were therefore restricted to descriptive patient-level trajectories. Descriptive statistical summaries are reported in the Supplementary Data without inferential intent.

The study complied with the Declaration of Helsinki, was approved by the Geneva Ethics Commission (2017-00640), and registered at ClinicalTrials.gov (NCT03397810).

## 3 Results

Between April 2019 and November 2021, 5 eligible patients with ATTRwt-CA were enrolled in the present study and treated per protocol. The mean age of the patients was 87 years, and 4 out of 5 were men. One patient had initiated tafamidis three months prior to study inclusion and continued treatment throughout the study period. A second patient started tafamidis six months after LD-RT, following completion of the primary follow-up. The study was terminated early owing to recruitment difficulties related to the COVID-19 pandemic, internal logistical challenges, and the evolving therapeutic landscape for ATTRwt-CA.

No grade ≥ 3 CTCAE v4.0 adverse effects were observed during the 6 months of follow-up related to LD-RT (see Supplemental data).

**Fig. 1:**
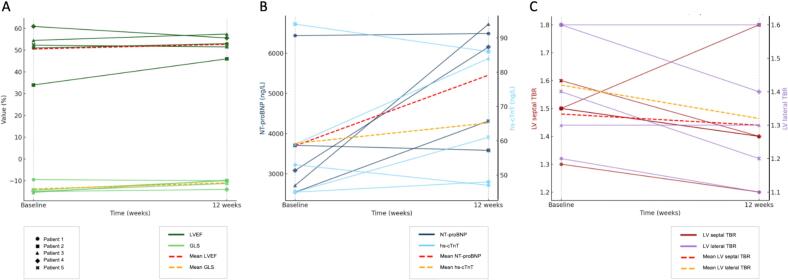
Changes in key cardiac parameters from baseline to 12 weeks in the five patients studied. A: Echocardiographic parameters (GLS, global longitudinal strain; LVEF, left ventricular ejection fraction); B: Cardiac biomarkers; C: Septal and lateral TBR (tissue to background ratio) estimating cardiac amyloid burden. Each patient is represented by a different symbol.

Individual patient-level data and changes from baseline to Week 12 for all five participants are summarized in Table 1 and Fig. 1.

**Table 1:** Patient-level changes in functional status, imaging parameters, and cardiac biomarkers from baseline to week 12 (patients receiving tafamidis are indicated with an asterisk). GLS: global longitudinal strain; LS: longitudinal strain; LV: left ventricle; LVEF: left ventricular ejection fraction; LVMi: left ventricular mass index; NYHA: New York Heart Association functional class; SF-36: Short Form–36 Health Survey; TBR: tissue-to-background ratio; 6MWT: six-minute walk test.

In the two patients receiving concomitant tafamidis during the study period, numerical improvements were observed across functional measures, cardiac biomarkers, and echocardiographic parameters. Among patients not receiving tafamidis, most parameters remained stable or showed numerical deterioration over the 12-week period, with heterogeneous individual trajectories across functional, imaging, and biomarker domains.

A numerical improvement in amyloid PET uptake ratio was observed in most treated patients, irrespective of tafamidis exposure. Conversely, despite heterogeneous individual changes, native T1 values and left ventricular mass index on CMR increased in most patients over the 12-week period, reflecting a predominantly non-improving directional pattern.

## 4 Discussion

To our knowledge, this is the first study to explore the feasibility and safety of LD-RT in patients with ATTRwt-CA. No LD-RT–related safety issues were reported during 6 months of follow-up. At 12 weeks, patient-level changes across functional, biomarker, and imaging domains were heterogeneous; however, a directional improvement in amyloid PET uptake ratio was observed in most patients, irrespective of tafamidis exposure.

Cardiac radiotherapy has been evaluated in other contexts, particularly ventricular arrhythmias, with acceptable safety profiles when carefully planned and delivered [18], [19]. Although these studies do not address amyloidosis, they provide indirect reassurance regarding feasibility. In our cohort, no early toxicity signal was identified. Long-term safety in ATTRwt-CA remains uncertain and requires extended follow-up, although given the advanced age of most affected patients, very late complications may be of lesser clinical relevance.

Analysis at the individual level revealed a directional decrease in amyloid PET uptake ratio. This modality was selected based on prior data suggesting its potential utility for ATTR-CA diagnosis and treatment monitoring [20]. Interpretation nevertheless requires caution. Baseline tracer uptake values were relatively modest, limiting the dynamic range for detecting change, and longitudinal PET variations were small in magnitude, potentially approaching measurement variability in such a limited sample. Although the quantification approach aligns with published reports, reproducibility and minimal detectable change data remain limited. Therefore, no definitive effect on myocardial amyloid burden can be inferred, although the observed directional pattern supports further evaluation in adequately powered studies.

In contrast, no clear clinical or functional benefit was observed. CMR parameters, including native T1 and LV mass index, generally showed no improvement at 12 weeks. Whether LD-RT influenced disease progression compared with natural history cannot be determined.

Several factors may explain the absence of clear efficacy signals. The radiation dose may have been insufficient to meaningfully reduce amyloid load; higher doses might increase efficacy but at the cost of toxicity. In addition, our elderly population with advanced disease, reflected by elevated baseline NT-proBNP levels, may have had limited potential for measurable short-term response. Finally, amyloid heterogeneity could play a role, as distinct amyloid protein subtypes with different structural properties may theoretically vary in their radiosensitivity.

## 5 Limitations of the study

This study has several important limitations. First, the small sample size, despite an initial target of 36 patients, substantially limits statistical power and restricts analyses to descriptive individual trajectories. Exploratory before–after statistical analyses are provided in the Supplementary Data but should not be interpreted as evidence of efficacy. Second, concomitant tafamidis exposure represents a potential confounder. One patient had initiated tafamidis three months prior to LD-RT and continued treatment throughout the study period, which may have influenced clinical and biomarker trajectories. A second patient started tafamidis only after completion of the 6-month follow-up and therefore does not confound the primary study outcomes. Third, the absence of a control group and the limited 6-month follow-up preclude conclusions regarding comparative efficacy or long-term safety. Finally, optimal dosing strategies for ATTRwt-CA radiotherapy remain to be defined.

## 6 Conclusion

In this small exploratory study of five patients with ATTRwt-CA, focused cardiac radiotherapy administered over five consecutive days was well tolerated, with no treatment-related safety concerns observed during 6 months of follow-up. Clinical and functional changes were heterogeneous and non-informative for efficacy, while amyloid PET showed a directional signal warranting further investigation.

## CRediT authorship contribution statement

**Damien Guijarro:** Writing – original draft, Formal analysis, Data curation. **Emmanuelle Massie:** Formal analysis, Data curation. **Thomas Zilli:** Writing – review & editing, Validation, Methodology, Investigation. **Anna Beale:** Writing – review & editing, Investigation. **Valentina Garibotto:** Writing – review & editing, Methodology, Investigation. **René Nkoulou:** Writing – review & editing, Methodology, Investigation. **Philippe Meyer:** Writing – review & editing, Validation, Supervision, Project administration, Methodology, Investigation, Funding acquisition, Conceptualization.

## Declaration of competing interest

The authors declare that they have no known competing financial interests or personal relationships that could have appeared to influence the work reported in this paper.
